# Mental State of Inpatients With COVID-19: A Computational Psychiatry Approach

**DOI:** 10.3389/fpsyt.2022.801135

**Published:** 2022-04-07

**Authors:** Mikhail Yu. Sorokin, Ekaterina I. Palchikova, Andrey A. Kibitov, Evgeny D. Kasyanov, Maria A. Khobeysh, Elena Yu. Zubova

**Affiliations:** ^1^The Integrative Pharmaco-Psychotherapy of Patients With Mental Disorders Department, V.M. Bekhterev National Medical Research Center for Psychiatry and Neurology, Saint Petersburg, Russia; ^2^The Geriatric Psychiatry Department, V.M. Bekhterev National Medical Research Center for Psychiatry and Neurology, Saint Petersburg, Russia; ^3^The Educational Department, V.M. Bekhterev National Medical Research Center for Psychiatry and Neurology, Saint Petersburg, Russia; ^4^The Translational Psychiatry Department, V.M. Bekhterev National Medical Research Center for Psychiatry and Neurology, Saint Petersburg, Russia

**Keywords:** SARS-CoV-2 infection, psychiatric disorder, mental status test, cluster analysis, COVID-19

## Abstract

**Background:**

The overload of healthcare systems around the world and the danger of infection have limited the ability of researchers to obtain sufficient and reliable data on psychopathology in hospitalized patients with coronavirus disease 2019 (COVID-19). The relationship between severe acute respiratory syndrome with the coronavirus 2 (SARS-CoV-2) infection and specific mental disturbances remains poorly understood.

**Aim:**

To reveal the possibility of identifying the typology and frequency of psychiatric syndromes associated with acute COVID-19 using cluster analysis of discrete psychopathological phenomena.

**Materials and Methods:**

Descriptive data on the mental state of 55 inpatients with COVID-19 were obtained by young-career physicians. Classification of observed clinical phenomena was performed with k-means cluster analysis of variables coded from the main psychopathological symptoms. Dispersion analysis with p level 0.05 was used to reveal the clusters differences in demography, parameters of inflammation, and respiration function collected on the basis of the original medical records.

**Results:**

Three resulting clusters of patients were identified: (1) persons with anxiety; disorders of fluency and tempo of thinking, mood, attention, and motor-volitional sphere; reduced insight; and pessimistic plans for the future (*n* = 11); (2) persons without psychopathology (*n* = 37); and (3) persons with disorientation; disorders of memory, attention, fluency, and tempo of thinking; and reduced insight (*n* = 7). The development of a certain type of impaired mental state was specifically associated with the following: age, lung lesions according to computed tomography, saturation, respiratory rate, C-reactive protein level, and platelet count.

**Conclusion:**

Anxiety and/or mood disturbances with psychomotor retardation as well as symptoms of impaired consciousness, memory, and insight may be considered as neuropsychiatric manifestations of COVID-19 and should be used for clinical risk assessment.

## Introduction

The neurotropic nature of severe acute respiratory syndrome with the coronavirus 2 (SARS-CoV-2) predetermines psychiatric disorders in some patients with coronavirus disease 2019 (COVID-19) (1–3). However, most publications on the psychological and mental impact of COVID-19 present the results of online and cross-sectional studies of the general population (4–6), some researches emphasize the healthcare service burden of clinics (7), and other studies present the post-recovery data of patients who have suffered the acute SARS-CoV-2 infection earlier (8–12). Even the clinical findings from previous coronavirus crises are mostly symptom- and dimension-oriented (13, 14).

The complex clinical picture and frequency of psychiatric syndromes in patients with current SARS-CoV-2 infection remain poorly understood (15, 16). A few studies present case reports of rare psychiatric conditions (17, 18). Some data were published about the existence of neurological disturbances in hospitalized patients with COVID-19 (19–21). Few studies are systematic assessments of the mental status of inpatients with COVID-19 (22, 23). These results are often obtained by non-psychiatric health professionals. At the same time, neuropsychiatric disorders are a COVID-19 death risk factor (23, 24), so they need to be diagnosed in a timely manner and appropriately treated. In this case, the lack of data on typical mental status variations in COVID-19 patients must be addressed because of the importance of this phenomenological information as a potential target for clinical screening and risk assessment by general practitioners.

At the same time, the extreme overload of healthcare systems around the world and the danger of infection have limited the ability of psychiatric researchers to obtain sufficient and reliable data on psychopathology in hospitalized patients with COVID-19. The relationship between severe infection and specific psychiatric syndromes remains to be explored. Back in the early days of psychiatry as a medical specialty, solving similar problems associated with syphilis and progressive paralysis took more than 100 years (25). Computational psychiatry is considered a promising methodology for assessing complex clinical events with a large number of factors and predictors that can lead to ambiguous clinical conditions in patients (26, 27). An important aspect of this approach is verification of the observed mental disturbances using certain pathogenetic indicators, such as inflammation and abnormalities of physiological functions (28).

The hypothesis of the study is as follows: nervous system damage caused by SARS-CoV-2 can have a variety of psychopathological manifestations in patients and must be associated with specific clinical parameters.

The aim of the study is as follows: to reveal the possibility of identifying the typology and frequency of psychiatric syndromes associated with acute COVID-19 using cluster analysis of discrete psychopathological phenomena.

## Method

**Figure 1:**
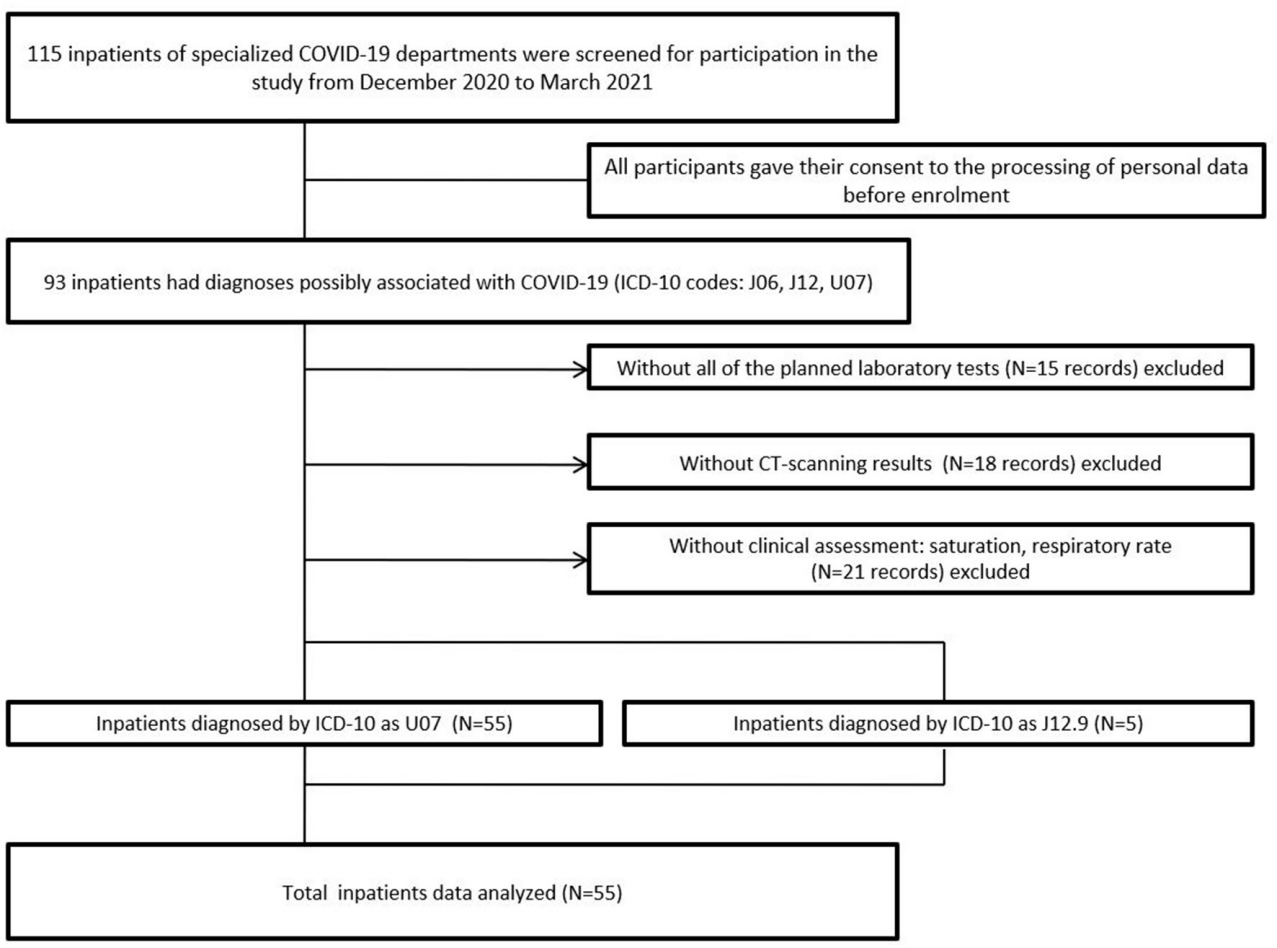
Flowchart of participant recruitment.

The assessment of the mental state of patients with COVID-19 requires specialized education and sufficient clinical practice of a physician. These requirements are unattainable in the real world of the COVID-19 crisis. During their mandatory general medicine practice in the northwest region of Russia, trainees of the National Medical Research Center for Psychiatry and Neurology obtained descriptive data on the mental state of 55 inpatients with COVID-19 (Figure 1). Between December 2020 and March 2021, resident psychiatrists, neurologists, and psychotherapists conducted semi-structured interviews with acute COVID-19 inpatients in infectious disease departments. Certain descriptors of psychopathological syndromes, laboratory results, and sociodemographic data of patients, as well as sources for their acquisition, are presented in Supplementary Table 1.

Young career physicians who had already completed their basic and advanced training courses in psychopathology provide enough quality in the process of data acquisition. To standardize the mental state assessment and to maximize inter-rater reliability, discrete psychopathological phenomena were pre-identified for raters. They used a scale from 0 to 1 point, where 0 = absence and 1 = presence of violations. The possible range of severity between 0 and 1 point should provide “artifact correction” during data acquisition, and k-means cluster analysis of quantitative variables coded from the main psychopathological symptoms allowed to perform classification of observed clinical phenomena. Quality control during data acquisition, artifact correction, and robust statistical algorithms are considered essential for computational technologies in psychiatry (29). The Student's *t*-test and the Mann–Whitney *U*-test with p-level of 0.05 were used to reveal cluster differences in parameters of inflammation and respiration function which were suggested as a physiological background of psychopathology in COVID-19 patients. Chi-square test was used for the assessment of cluster differences in socio-demographic parameters and presence of comorbidities. Clinical parameters of the patients were collected on the basis of the original medical records. Descriptions of subgroups were presented in means M[SD] or medians Me(IQR) depending on the results of distribution normality tests (Kolmogorov–Smirnov K-test). IBM SPSS Statistics (RRID:SCR_019096) was used.

The study design was controlled by the independent ethical committee. It was in conformity with the Helsinki Declaration and the standard of good clinical practice (GCP). It included collection of anamnestic socio-demographic data and clinical parameters based on the original medical records after the patients signed a voluntary informed consent, and their current mental state was tested.

The inclusion criteria were the following: (1) ability to read and understand and readiness to sign a voluntary informed consent to take part in the study; (2) a hospitalization due to COVID-19 diagnosis; and (3) ability to fulfill the study procedures.

The non-inclusion criteria were the following: (1) extremely high severity of the current condition with insufficient respiratory function and (2) age <18 years. Exclusion criterion was the following: refusal to comply with the study procedures at any stage of the study.

## Results

The sample of patients consisted of 21 men and 34 women, with a mean age of 51.5 [20.9] years. Higher and not completed higher education was characteristic of 30 patients (54.5%), secondary education of 11 patients (20.0%), and primary education of 14 patients (25.5%). The majority of the sample of patients were married people−33 (60%), and the smaller share was single persons−21 patients (38.2%). Also, the majority of the patients studied or worked full time−31 (56.4%), and the smaller share was unemployed−23 (41.8%). Data about the marital status for one patient (1.8%) and about the occupation for another one (1.8%) were missing (Supplementary Table 2). The most prevalent comorbidities were cardiovascular disorders −11 patients (20.0%), then endocrine disorders−6 (10.6%), gastrointestinal−5 (9.1%), and respiratory−2 (3.6%); renal and neurological disorders were the rarest—in 1 patient (1.8%) for each comorbidity. The mean percentage of lung lesions according to computed tomography data was 20.1% [19.1], and saturation lower than 95% was characteristic of 16 patients.

**Figure 2:**
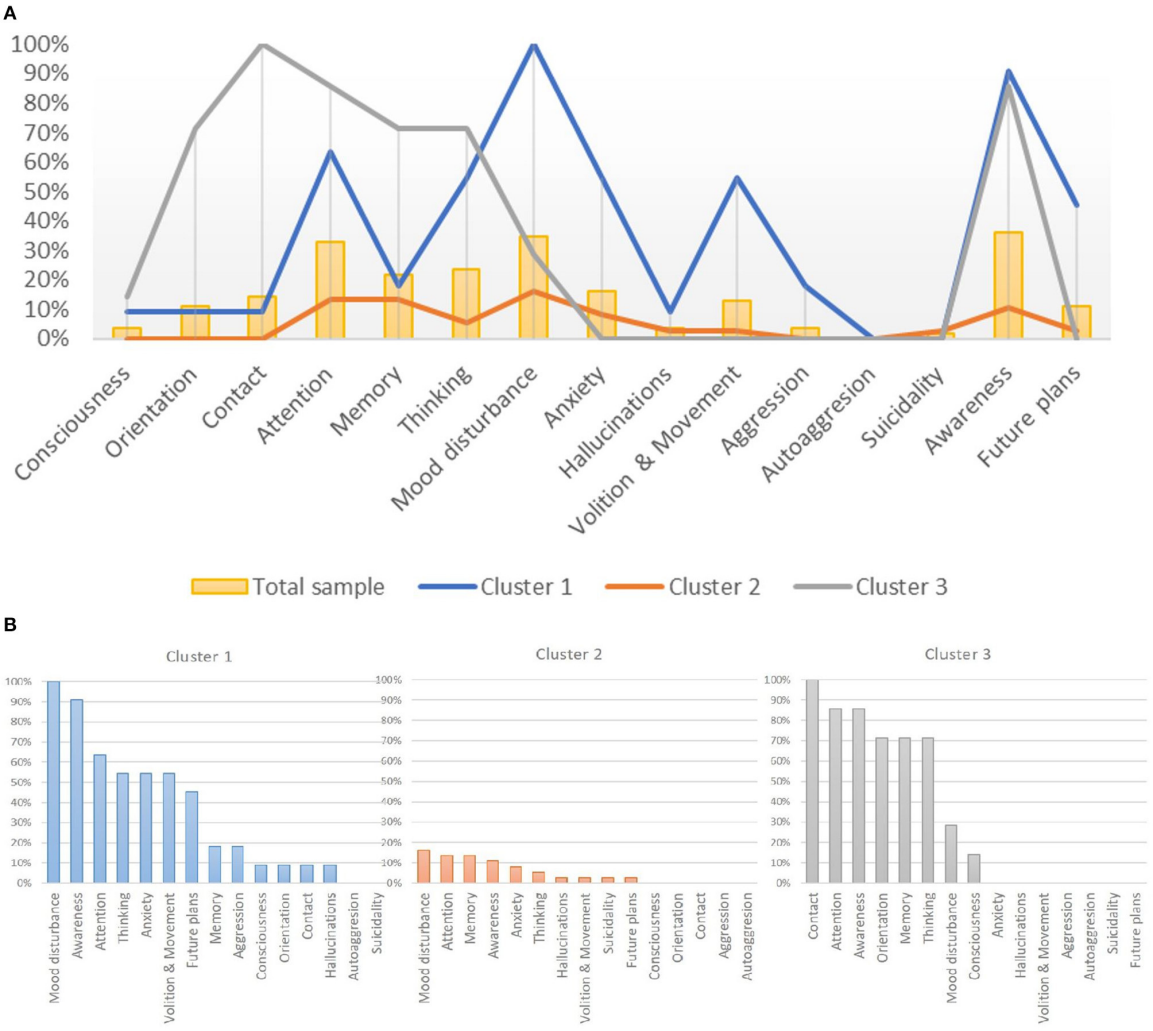
**(A)** The frequency of psychopathological syndromes in patients with COVID-19 infection within three defined clusters. **(B)** Psychopathological profiles of inpatients with COVID-19 infection in each determined cluster.

Three resulting clusters of patients were identified (without differences in gender and somatic and mental comorbidities) (Figure 2A). The first cluster [*n* = 11 (20%)] was of patients with anxiety; disorders of fluency and tempo of thinking, mood, attention, and motor-volitional sphere; reduced insight; and pessimistic plans for the future. The second cluster [*n* = 37 (67%)] was of patients without psychopathology. The third cluster [*n* = 7 (13%)] was of patients with disorientation; disorders of memory, attention, fluency, and tempo of thinking; and reduced insight (Figure 2B).

Representatives of cluster 1, in comparison with cluster 2 (without mental disturbances), had more lung lesions according to computed tomography: 20% (34) vs. 15% (18), *p* = 0.018. There were no significant differences in saturation, respiratory rate, and other laboratory parameters, as well as in age between patients from cluster 1 and cluster 2.

Other patients with mental abnormalities (cluster 3) were older: 76.9 [14.7] vs. healthy patients (cluster 2) 50.9 [17.8], *p* = 0.001, as well as vs. patients with anxiety and mood disturbances (cluster 1) 60.9 [24.3], *p* = 0.027. Cluster 3 patients, in comparison with cluster 2 (patients without mental abnormalities), were clinically different by a more severe course of the disease based on the results of laboratory and instrumental methods: a higher percentage of lung damage [31% (35) vs. 15% (18), *p* < 0.001]; higher level of C-reactive protein [126 mg/L (236) vs. 10 mg/L (21), *p* < 0.001]; lower saturation [89% (13) vs. 97% (4), *p* < 0.001]; and higher respiratory rate [21 (6) vs. 18 (4), *p* < 0.001].

Patients from cluster 3 vs. cluster 1 clinically differed: a higher percentage of lung lesions on computed tomography [31% (35) vs. 25% (34), *p* = 0.029], higher C-reactive protein level [126 mg/L (236) vs. 16 mg/L (88), *p* < 0.001], lower saturation [89% (13) vs. 95.5% (4), *p* = 0.005], higher respiratory rate [21/min (6) vs. 19/min (7), *p* = 0.035], and lower platelet count [139 ^*^ 10^9^/L (129) vs. 322 ^*^ 10^9^/L (129), *p* = 0.006].

## Discussion

To our knowledge, this is the first study performed using the computational psychiatry approach to assess the presence and typology of psychopathological syndromes in patients with acute COVID-19. The hypothesis of the study was confirmed: differences in the presence of psychopathology and the development of a certain type of impaired mental state were associated with specific clinical and laboratory parameters of patients. The combined representation of anxiety and/or mood disturbances with psychomotor retardation was characteristic of 20% of inpatients with acute COVID-19. Symptoms of impaired consciousness and memory, combined with impaired insight, were present in 13% of the sample.

The study had several limitations. Firstly, patients in extremely severe current condition with insufficient respiratory function were not included in the study, although they could have more pronounced mental disturbances. The second limitation was the small size of the sample due to the limited access to COVID-19 patients. Thirdly, standardized psychiatric diagnostic methods and tests or specific surveys (30) were not used because of the lack of time and acute infection process in the study participants. The structure of mental state examination traditionally used in Russian medical praxis founded mainly in German psychiatry was implied (31). The list of psychopathology dimensions used for assessment in the study is matched to British Medical Association guidance (2004). The slight modification of this list was made in accordance with the basic course in psychopathology (32). The fourth limitation was the issue of reliability of assessment performed by a general physician without psychiatric license. To minimize this possible weakness, in the study residents in psychiatry, neurology, and psychotherapy performed the assessment of the mental state within their competencies due to not only basic but advanced courses in psychopathology. This made data acquisition robust enough for further computational processing.

The results of the study should be used for better risk assessment of people with coronavirus infection and prediction of neuropsychiatric consequences as a marker of a more unfavorable course of the disease.

## Data Availability Statement

The raw data supporting the conclusions of this article will be made available by the authors, without undue reservation.

## Ethics Statement

The studies involving human participants were reviewed and approved by Independent Ethics Committee of V.M. Bekhterev National Medical Research Center for Psychiatry and Neurology. The patients/participants provided their written informed consent to participate in this study.

## Author Contributions

EZ and MS: conceptualization of the study, goals and aims, and resources. MS, EP, and AK: investigation. MS and AK: methodology, statistics, and writing (original draft). MS and MK: writing (review and editing). MS, EP, and EK: project administration. All authors read and approved the final version of the manuscript.

## Conflict of Interest

The authors declare that the research was conducted in the absence of any commercial or financial relationships that could be construed as a potential conflict of interest.

## Publisher's Note

All claims expressed in this article are solely those of the authors and do not necessarily represent those of their affiliated organizations, or those of the publisher, the editors and the reviewers. Any product that may be evaluated in this article, or claim that may be made by its manufacturer, is not guaranteed or endorsed by the publisher.
