# Supplementary material for: Mental State of Inpatients With COVID-19: A Computational Psychiatry Approach
Source: Front Psychiatry. 2022 Apr 7;13:801135. doi: 10.3389/fpsyt.2022.801135 (PMC9021726; doi:10.3389/fpsyt.2022.801135)
Supplement: Supplementary file 1 [file Table_1.DOCX]

| Patient ID: |  |
| --- | --- |
| Date: |  |
| **DATA COLLECTED FROM MEDICAL RECORDS (IN ACCORDANCE WITH THE ROUTINE MEDICAL PRACTICE IN CERTAIN HOSPITAL):** | |
| Age: |  |
| Sex: | *Male* |
|  | *Female* |
| Education: | *Secondary general* |
|  | *Secondary professional* |
|  | *Incomplete higher* |
|  | *Complete higher* |
| Occupation: | *Not working/studying* |
|  | *Working/Studying* |
| Marital status: | *Single* |
|  | *Married* |
| Concomitant mental disorders: | *None* |
|  | *Depressive disorders* |
|  | *Anxiety disorders* |
|  | *Schizophrenia spectrum disorders* |
| Neurological comorbidities: | *None* |
|  | *Any* |
| Cardiovascular comorbidities: | *None* |
|  | *Any* |
| Pulmonary comorbidities: | *None* |
|  | *Any* |
| Respiratory comorbidities: | *None* |
|  | *Any* |
| Renal and urogenital comorbidities: | *None* |
|  | *Any* |
| Endocrine comorbidities: | *None* |
|  | *Any* |
| Confirmation of the diagnosis of COVID-19 | *PCR* |
|  | *CT* |
|  | *ARVI* |
|  | *Healthy* |
| *In the case of several answers, the most reliable criterion is selected (PCR-CT-ARVI (in decreasing order of reliability))* | |
| Percentage of lung lesion according to CT scans (%): |  |
| *For bilateral lesions with different values, the average percentage is indicated* | |
| C-reactive protein level (mg/l) |  |
| Saturation (%) |  |
| Respiratory rate (breaths/minute) |  |
| Leukocyte count (*10^9/l) |  |
| Lymphocyte count (*10^9/l) |  |
| Platelet count (*10^9/l) |  |
| **DATA COLLECTED DURING SEMI-STRUCTURED INTERVIEW** | |
| Mental state: phenomenological depiction of certain descriptors | *consciousness disturbances, orientation disturbances, productivity of contact with a physician, attention, memory, difficulties of thinking, mood disturbance, anxiety symptoms, all kinds of hallucinations, volition and movement disturbances, physical and/or verbal aggression, auto-aggressive behaviour, suicidality, awareness of mental disturbances, realistic plans for the future.* |

**Supplement Table 1.** Survey form.
