# Supplementary material for: Mental State of Inpatients With COVID-19: A Computational Psychiatry Approach
Source: Front Psychiatry. 2022 Apr 7;13:801135. doi: 10.3389/fpsyt.2022.801135 (PMC9021726; doi:10.3389/fpsyt.2022.801135)
Supplement: Supplementary file 2 [file Table_2.DOCX]

| Assessed characteristics | Presence in a certain cluster, n (%) | | | Criterion of statistical difference |
| --- | --- | --- | --- | --- |
|  | Cluster 1 | Cluster 2 | Cluster 3 |  |
| Sex | | | | |
| Male | 6 (54,5%) | 14 (37,8%) | 1 (14,3%) | χ^2^(2)=2,943, p=0,23 |
| Female | 5 (45,5%) | 23 (62,2%) | 6 (85,7%) |  |
| Education | | | | |
| Secondary general | 5 (45,5%) | 8 (21,6%) | 1 (14,3%) | χ^2^(6)=6,313, p=0,389 |
| Secondary professional | 2 (18,2%) | 6 (16,2%) | 3 (42,9%) |  |
| Incomplete higher | 1 (9,1%) | 5 (13,5%) | 0 (0%) |  |
| Complete higher | 3 (27,3%) | 18 (48,6%) | 3 (42,9%) |  |
| Occupation | | | | |
| Not working/studying | 6 (54,5%) | 12 (32,4%) | 5 (83,3%) | χ^2^(2)=6,278, p=0,043 |
| Working/Studying | 5 (45,5%) | 25 (67,6%) | 1 (16,7%) |  |
| Marital status | | | | |
| Single | 3 (27,3%) | 15 (41,7%) | 3 (42,9%) | χ^2^(2)=0,788, p=0,674 |
| Married | 8 (72,7%) | 21 (58,3%) | 4 (57,1%) |  |
| Comorbid mental disorders | | | | |
| None | 8 (72,7%) | 34 (91,9%) | 7 (100%) | χ^2^(6)=9,051, p=0,171 |
| Depressive disorders | 1 (9,1%) | 0 (0%) | 0 (0%) |  |
| Anxiety disorders | 1 (9,1%) | 0 (0%) | 0 (0%) |  |
| Schizophrenia spectrum disorders | 1 (9,1%) | 3 (8,1%) | 0 (0%) |  |
| Neurological comorbidities | | | | |
| None | 10 (90,9%) | 37 (100,0%) | 7 (100%) | χ^2^(2)=4,074, p=0,130 |
| Any | 1 (9,1%) | 0 (0%) | 0 (0%) |  |
| Cardiovascular comorbidities | | | | |
| None | 8 (72,7%) | 29 (78,4%) | 7 (100%) | χ^2^(2)=2,174, p=0,337 |
| Any | 3 (27,3%) | 8 (21,6%) | 0 (0%) |  |
| Pulmonary comorbidities | | | | |
| None | 11 (100%) | 35 (94,6%) | 7 (100%) | χ^2^(2)=1,010, p=0,604 |
| Any | 0 (0%) | 2 (5,4%) | 0 (0%) |  |
| Respiratory comorbidities | | | | |
| None | 9 (81,8%) | 34 (91,9%) | 7 (100%) | χ^2^(2)=1,843, p=0,398 |
| Any | 2 (18,2%) | 3 (8,1%) | 0 (0%) |  |
| Renal and urogenital comorbidities | | | | |
| None | 11 (100%) | 36 (97,3%) | 7 (100%) | χ^2^(2)=0,495, p=0,781 |
| Any | 0 (0%) | 1 (2,7%) | 0 (0%) |  |
| Endocrine comorbidities | | | | |
| None | 9 (81,8%) | 33 (89,2%) | 7 (100%) | χ^2^(2)=1,456, p=0,483 |
| Any | 2 (18,2%) | 4 (10,8%) | 0 (0%) |  |

Supplement Table 2. Sociodemographic characteristics and data on the comorbidities in determined clusters.
